# Operator volumes and salvage index in AMI

**DOI:** 10.1186/1532-429X-14-S1-P22

**Published:** 2012-02-01

**Authors:** Daniel J Dooley, Michael Kern, Ashish Haryani, Manuel A Gonzalez, Rebecca Torguson, Ron Waksman, Gaby Weissman, Anthon Fuisz

**Affiliations:** 1 Washington Hospital Center, Washington, DC, USA; 2 Georgetown University, Washington, DC, USA

## Background

Cardiac MR (CMR) is a useful diagnostic tool for the evaluation of myocardial infarction. While several previous studies have assessed the effect of operator volumes on outcome following coronary angioplasty, none have utilized salvage index measured by CMR as a primary endpoint. Prior studies relying on major adverse cardiac events as endpoints required very large sample sizes for sufficient power. The prognostic ability of CMR can, in theory, reduce the minimum necessary sample size, as recent research has provided evidence of CMR as a reliable, reproducible method for determining infarct size and AAR and salvage index via T2-weighted imaging.

### Hypothesis

CMR imaging will show improved salvage index in patients who undergo primary PCI with high-volume physicians.

## Methods

This single-center study included 28 patients with STEMI treated with primary PCI. Ten cardiologists with varying annual PCI volumes were involved. Both T2 and late gadolinium enhanced CMR were performed in patients within 3+/-2 days of STEMI, and CMR imaging was repeated in each patient after 30 days.

## Results

Baseline characteristics of the patients involved in the study included 22 of 28 (78.6%) male, age of 52.1 +/-12.2 years., hypertension in 8 of 28 (28.6%), and 6 of 28 (21.4%) with diabetes. Area at risk (AAR) was determined by T2 weighted black blood images and myocardial scarring was determined by late gadolinium enhanced (LGE) images. Mean salvage index was calculated after initial imaging for lower volume cardiologists (7.02% +/-3.52%) and higher volume cardiologists (7.58% +/-7.75%), which were not significantly different when compared using student’s t-test (p=0.8384). When repeated 30 days later, mean salvage index was 0.48% +/-9.64% for lower volume cardiologists and 1.22% +/-6.10% for higher volume cardiologists (p=0.80656). The annual operator volumes of each cardiologist ranged from 46 to 565 in the lower volume group, and from 864 to 1647 in the higher volume group. There was no statistically significant difference between baseline characteristics of patients (age, gender, lipid profile, hypertension or diabetes) treated by either the lower volume cardiologists or the higher volume cardiologists.

## Conclusions

Larger operator volumes are not associated with a significant difference in myocardial salvage index when comparing two groups of cardiologists with higher annual volumes and lower annual volumes.

## Funding

Medstar Cardiovascular Research Institute.

